# A Giant Aneurysm of Vieussens’ Arterial Ring With Pulmonary Artery Fistula

**DOI:** 10.1016/j.atssr.2024.05.008

**Published:** 2024-06-04

**Authors:** Kosuke Nakata, Shuji Moriyama, Takashi Yoshinaga, Takehiro Ishimaru, Hideki Doi, Toshiyuki Matsumura, Toshihiro Fukui

**Affiliations:** 1 Department of Cardiovascular Surgery, Kumamoto Rosai Hospital, Yatsushiro, Kumamoto, Japan; 2 Department of Cardiovascular Surgery, Kumamoto University Hospital, Kumamoto, Japan; 3 Department of Cardiology, Kumamoto Rosai Hospital, Yatsushiro, Kumamoto, Japan

## Abstract

A 79-year-old woman presented with a systolic murmur and dyspnea on exertion. Transthoracic echocardiography and multidetector-row computed tomography revealed a giant aneurysm in an abnormal vessel known as Vieussens’ arterial ring (VAR). A pulmonary artery VAR fistula was also observed. Cardiac catheterization revealed a high pulmonary-to-systemic flow ratio (Qp/Qs = 2.1). We ligated the VAR, resected the aneurysm, and closed the fistula using a bovine pericardial patch. The patient’s postoperative clinical course was uneventful.

Vieussens’ arterial ring (VAR) is a rare coronary abnormality in adults. Coronary artery aneurysms (CAAs) complicated by pulmonary artery (PA) fistulas are unusual; aneurysms in the VAR are very rare. We found 17 case reports on PubMed for the term “Vieussens aneurysm.” However, no reports were found on the surgical treatment of giant CAA found full-length VAR with a PA fistula. We report such a case in this study.

A 79-year-old woman was referred to our hospital for the examination of varicose veins in the lower extremities. Cardiac auscultation revealed a systolic murmur at the 3 left sternal borders (Levine III-IV/IV). Transthoracic echocardiography showed CAAs. Multidetector-row computed tomography showed beaded giant CAAs between the origin of the left coronary artery (LCA) and right coronary artery (RCA) and a PA fistula (Figure 1). Cardiac catheterization revealed a high pulmonary-to-systemic flow ratio (Qp/Qs = 2.1) (Figure 2). Therefore, we decided to perform surgery. We approached the patient via a median sternotomy. An abnormal vessel, the VAR, was observed in the anterior PA. A giant CAA was found in the full-length VAR (Figure 3). The thrill was palpated at the PA root. The intraoperative Qp/Qs was 3.3. Intraoperative echocardiography revealed a coronary PA fistula anterior to the PA root. Before ligating the VAR, no electrocardiogram changes were observed after clamping. During cardiac arrest, we transected the main PA trunk and ligated the VAR on the LCA side. We resected the CAA except where it was attached to the myocardium. A fistula connected to the CAA on the luminal side of the PA trunk was identified and closed using a bovine pericardial patch (Edwards Life Sciences) on the PA’s luminal side (Figure 3). The absence of other fistulae was confirmed, and the main trunk of the PA was reconstructed. The patient’s postoperative clinical course was uneventful. Postoperative multidetector-row computed tomography revealed no obvious coronary connections to the PA. Postoperative transthoracic echocardiography revealed no asynergy or decreased ejection fraction. The patient was transferred from the hospital for rehabilitation 28 days after surgery.

**Figure 1:**
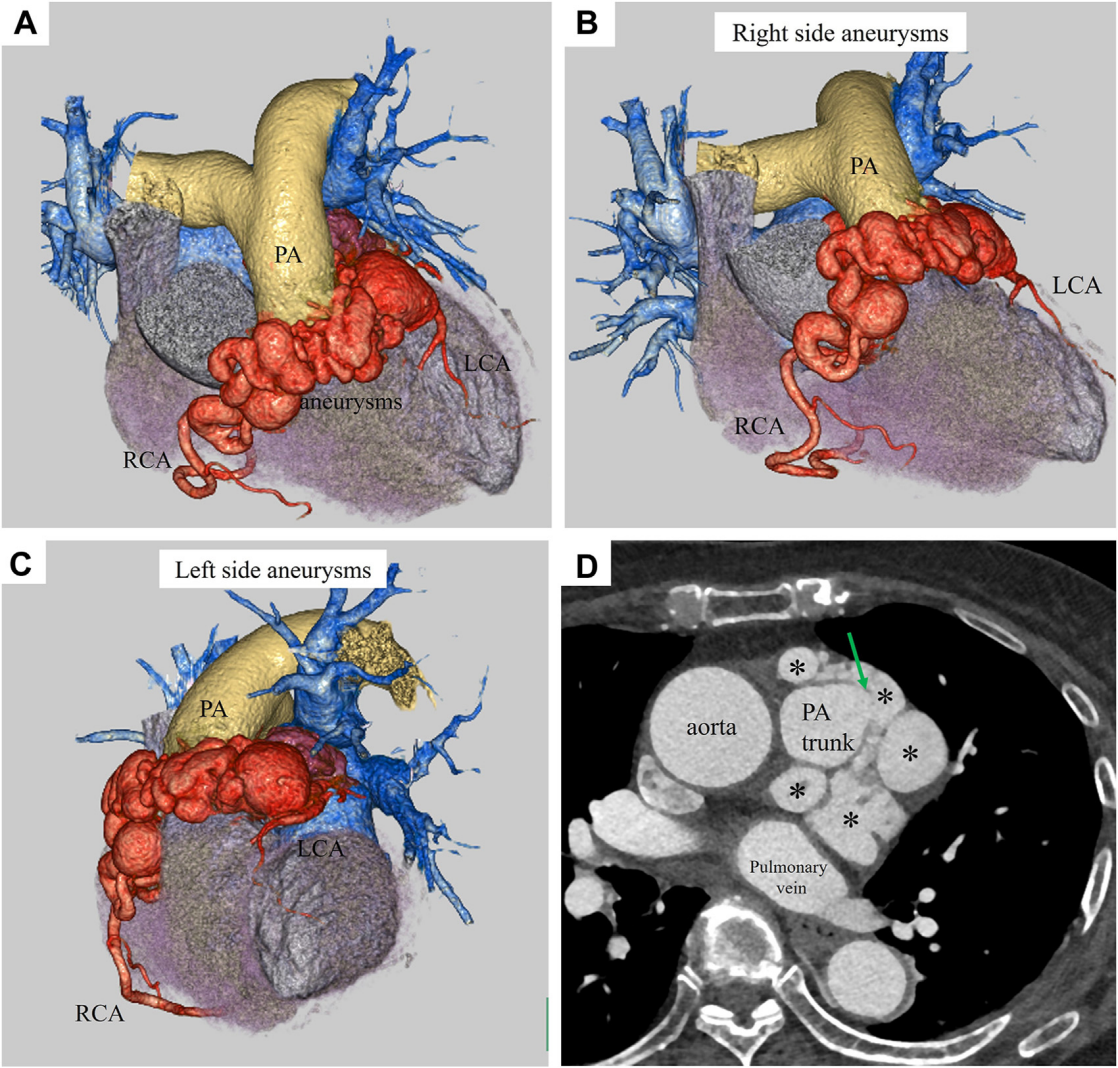
Preoperative multidetector-row computed tomography shows beaded giant coronary artery aneurysms (GCAA) in front of the pulmonary artery (PA) trunk. GCAAs (asterisk) connect the left coronary artery (LCA) with the right coronary artery (RCA). A GCAA-PA fistula is evident (green arrow).

**Figure 2:**
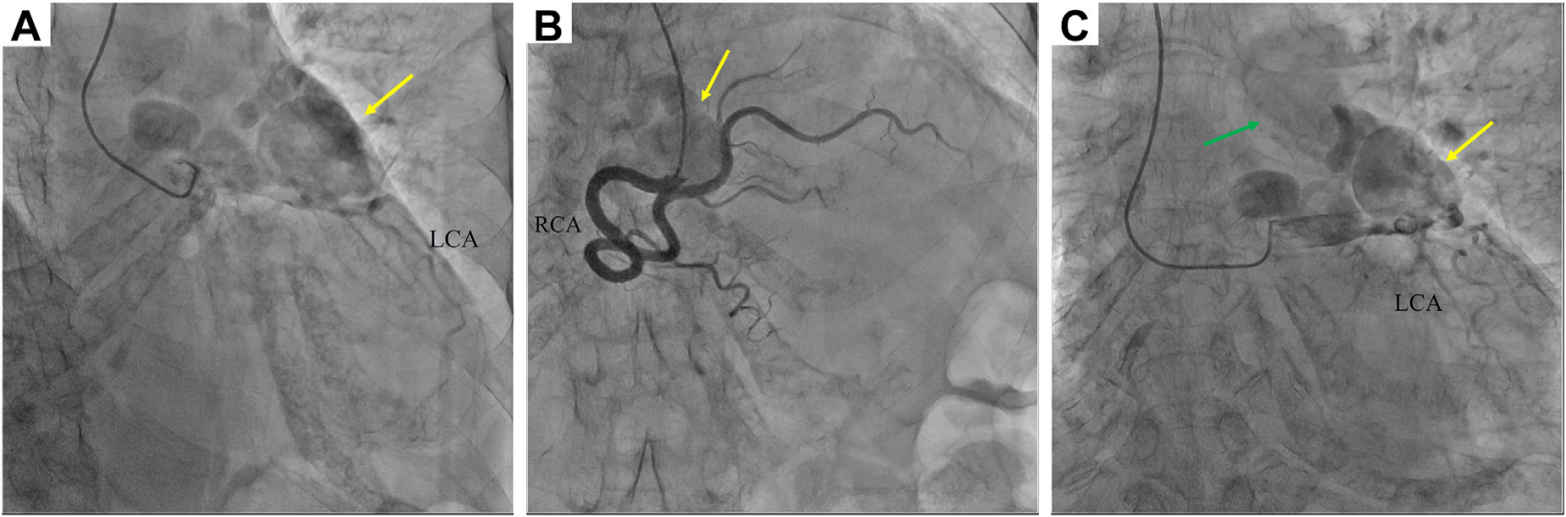
(A) and (C) show left coronary artery injected with X-ray contrast dye to show aneurysm. (B) shows right coronary angiography. Coronary angiography reveals a beaded giant coronary artery aneurysm (yellow arrow). Contrast enhancement of the pulmonary artery is observed (green arrow). (LCA, left coronary artery; RCA, right coronary artery.)

**Figure 3:**
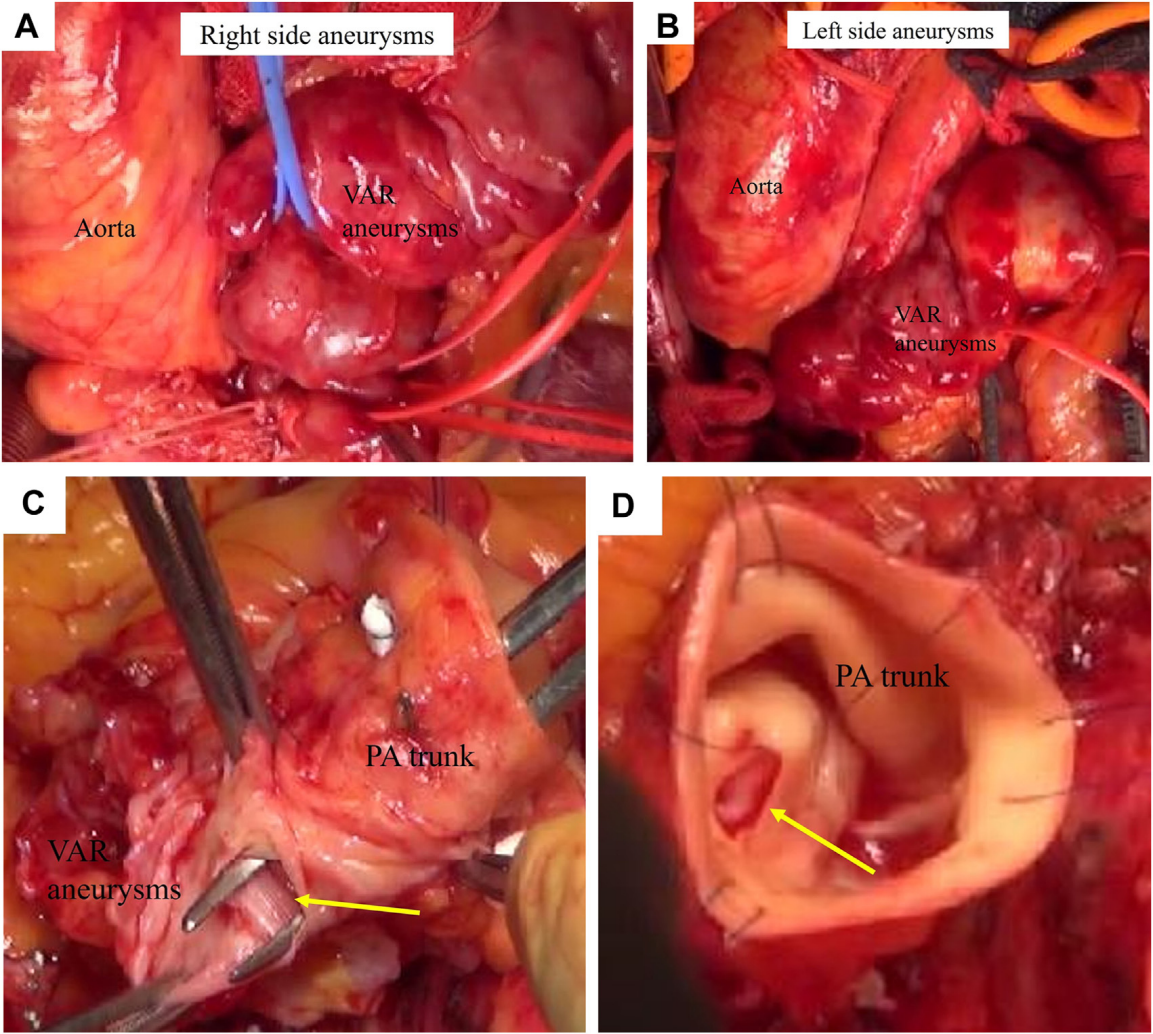
An abnormal vessel, Vieussens’ arterial ring (VAR), shown in front of the pulmonary artery (PA) trunk and aortic root, is aneurysmal and tortuous, connecting the left coronary artery and right coronary artery. Some aneurysms are approximately 30 mm. A fistula (yellow arrow) connects the PA trunk and VAR (approximately 10 mm).

## Comment

In 1706, Raymond de Vieussens reported the VAR, a vessel connecting the LCA and RCA.^1^ The VAR is thought to be a remnant of an embryologic vessel.^2^ Doğan and associates^1^ reported a VAR detection rate of 0.319% for 3443 consecutive coronary computed tomography angiographies. When stenosis develops in LCA or RCA, VAR acts as a collateral vessel.^1^^,^^3^ Symptomatic VAR is rare; most cases are caused by a fistula between the VAR and PA.^3^

A VAR-PA fistula is a subtype of coronary artery fistula (CAF).^3^ The frequency of CAF is estimated at 0.002%.^4^ The etiology of CAF is mainly congenital; secondary causes include infection, iatrogenicity, and trauma.^4^ Most patients are asymptomatic; CAF is diagnosed by chance during examinations. In the present case, auscultation led to a diagnosis of CAF. The loudest point of a murmur varies depending on the fistula’s location.^4^ If a fistula is present in the PA, as in this case, the loudest point of the cardiac murmur is at the left sternal border. Vasodilation and aneurysms are caused by persistent high blood flow into a vessel because of the fistula. Thus, a VAR-PA fistula produces a secondary VAR aneurysm.^3^

A CAA is defined as being 1.5 times larger than the normal or maximum diameter.^5^ CAAs are estimated to occur in 0.3% to 5% of patients undergoing coronary angiography.^5^ However, the pathogenesis of CAA is not fully understood. One hypothesis is that stenosis causes a turbulent blood flow, resulting in CAA in aneurysms near the coronary stenosis.^6^ Another hypothesis is that the rupture of intimal plaques causes ulceration, promoting vascular dilation.^5^ Although no criteria are definite, a vascular diameter >20 mm or 4 times the normal diameter is called a giant CAA.^7^ Their incidence is 0.02%, significantly lower than that of CAA.^7^ Jha and colleagues^8^ reported that 14 of 28 giant CAA cases were caused by atherosclerosis, and 2 congenital cases were found.

The treatments for these diseases have no established guidelines. Thus, the management plan is based on the individual symptoms or state of the patient. The treatments include medical therapy, percutaneous coronary intervention, and surgery. The surgical indications for CAA are a concomitant cardiac surgical procedure, multivessel disease, left main coronary artery involvement, mechanical complications, and multiple CAAs.^7^

Our patient had no history of cardiac diseases, including Kawasaki disease, and no cardiovascular anomalies were found. However, VAR and VAR-PA fistulas were observed, suggesting that congenital factors and fistulas caused the giant CAA. She complained of dyspnea on exertion, and the L-R shunt increased Qp/Qs. During surgery, we harvested the skeletonized left internal thoracic artery for intraoperative contingencies, including coronary artery injury and myocardial ischemia. We ligated the VAR entry under heartbeat to confirm that no electrocardiogram change occurred due to clamping the VAR. The surgery was performed with a cardiopulmonary bypass to avoid intraoperative circulatory collapse. Aneurysms around the LCA were hidden behind the pulmonary trunk. We transected the pulmonary trunk because they were difficult to approach. After patch closure of the fistula, the absence of other blood flow into the PA was confirmed using selective antegrade cardioplegia. After resuming the patient’s heartbeat, we confirmed that no turbulence was present in the PA on transesophageal echocardiography and transpulmonary-arterial echocardiography. Coronary artery bypass grafting was not performed since no myocardial ischemia was evident. The patient’s postoperative clinical course was uneventful. Transthoracic echocardiography in the third postoperative month showed no asynergy or reduction in the left ventricular ejection fraction. This very rare case of successful surgical treatment of a giant CAA of full-length VAR with a PA fistula can inform clinicians managing such similar cases.

## References

[1] Doğan N., Dursun A., Özkan H. (2019). Vieussens’ arterial ring: a rare coronary variant anatomy. Diagn Interv Radiol.

[2] Klein L.W., Campos E.P. (2019). The embryologic origin of Vieussens’ ring. J Invasive Cardiol.

[3] Ge C., Mao D., Ni J. (2021). Luminal diameter ratio of Vieussens’ arterial ring is valuable in determining appropriate clinical management for patients with pathologic Vieussens’ arterial ring. Int J Cardiol.

[4] Buccheri D., Chirco P.R., Geraci S., Caramanno G., Cortese B. (2018). Coronary artery fistulae: anatomy, diagnosis and management strategies. Heart Lung Circ.

[5] Vadalà G., Di Caccamo L., Alaimo C. (2022). Coronary arteries aneurysms: a case-based literature review. Diagnostics (Basel).

[6] Holman E., Peniston W. (1955). Hydrodynamic factors in the production of aneurysms. Am J Surg.

[7] Pham V., Hemptinne Q., Grinda J.M., Duboc D., Varenne O., Picard F. (2020). Giant coronary aneurysms, from diagnosis to treatment: a literature review. Arch Cardiovasc Dis.

[8] Jha N.K., Ouda H.Z., Khan J.A., Eising G.P., Augustin N. (2009). Giant right coronary artery aneurysm- case report and literature review. J Cardiothorac Surg.

